# Mast Cells Control the Bacterial Burden and Grain Formation in Murine Experimental Actinomycetoma Induced by *Nocardia brasiliensis*

**DOI:** 10.20411/pai.v11i2.1003

**Published:** 2026-07-16

**Authors:** Tomás Alejandro Suárez-Vázquez, Nallely López-López, Manuel Mejía-Torres, Adrián G Rosas-Taraco, Raquel Garza-Guajardo, Yatsiri G. Meneses-Preza, Bibiana Patricia Ruiz-Sánchez, Rommel Chacón-Salinas, Mario César Salinas-Carmona

**Affiliations:** 1 Servicio de Inmunología, Universidad Autónoma de Nuevo León, Hospital Universitario Jose E. Gonzalez, Monterey, Nuevo León, Mexico; 2 Servicio de Patología, Universidad Autónoma de Nuevo León, Hospital Universitario José E. González, Monterrey, Nuevo León, Mexico; 3 Departamento de Inmunología, Instituto Politécnico Nacional, Escuela Nacional de Ciencias Biológicas, Mexico

**Keywords:** Mast Cells, Actinomycetoma, *Nocardia Brasiliensis*, Wsh Mice, Chronic Bacterial Infection

## Abstract

**Background:**

The immunopathogenesis of actinomycetoma, a chronic granulomatous infection caused primarily by *Nocardia brasiliensis*, involves poorly defined mechanisms sustaining its characteristic cyclical inflammation.

**Methods:**

Using MCdeficient *Kit^W-sh/W-sh^* mice, this study investigated the role of mast cells (MCs) in a murine model of actinomycetoma. Clinical progression, bacterial burden, histopathology, and lesional leukocyte infiltration were analyzed at 30 and 72 days postinfection, and bone marrowderived MCs were challenged *in vitro* with *Nocardia brasiliensis* to assess activation.

**Results:**

MC-deficient mice exhibited a significant increase in bacterial load concomitant with attenuated late-phase (72-day) inflammation, characterized by reduced lesional neutrophilia and lymphocytic infiltration. Furthermore, MC absence disrupted the characteristic morphology of bacterial grains. *In vitro, N. brasiliensis* induced potent MC degranulation and pro-inflammatory cytokine release.

**Conclusions:**

These data demonstrate that MCs are directly activated by *N. brasiliensis* and contribute to a delayed protective immune response, modulating late-stage inflammatory containment and bacterial clearance in experimental mycetoma.

## Graphical abstract

**Figure:**
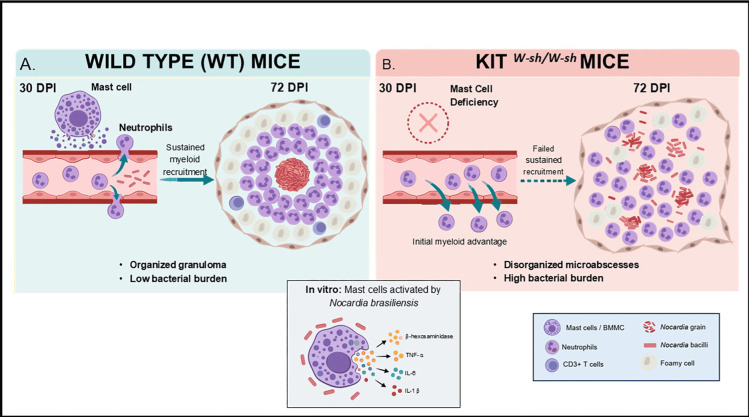

## INTRODUCTION

In Mexico, *Nocardia brasiliensis* is the primary cause of clinical actinomycetoma, a chronic and tissue-destructive infection that mostly affects immunocompetent individuals [1]. The general immunopathology of this condition was previously described by Salinas-Carmona *et al* and includes intense inflammation, abscess formation, and the presence of draining fistulas and granulomas [2]. Interestingly, the nature of this infectious disease has been described as a continuously relapsing disease, by the predominance of neutrophil infiltrates and the increased expression of exhausting lymphocyte markers [3, 4]. Actinomycetoma is associated with relapsing inflammation and requires the continuous activation of several resident cells through the detection of damage- and pathogen-associated molecular patterns, where mast cells stand out for their intrinsic inflammatory capabilities [5]. Beyond allergy, mast cells have been proven to be critical for acute viral and bacterial infections, and surprisingly even for chronic bacterial infections such as tuberculosis [5–7]. Therefore, we sought to evaluate the role of mast cells during *N. brasiliensis* infection, a role that has not been previously addressed. To gain insight into this matter, we analyzed the effect of mast cell deficiency on the formation of experimental actinomycetoma in mice using mast cell-deficient *Kit^W-sh/W-sh^* mice. In this study, we showed that mast cell deficiency has a delayed role in established actinomycetoma, manifested through poor bacterial control, decreased inflammation, and morphological alterations in bacterial grain formation compared to mast cell-competent mice. Furthermore, we noticed that *N. brasiliensis* was able to activate mast cells *in vitro*.

## METHODS

### Mice and Experimental Actinomycetoma

Female wild-type (WT) and mast cell-deficient *Kit^W-sh/W-sh^* mice, both on a C57BL/6 background, aged 12 weeks and weighing 25 ± 2 g, were used in the experiments. Mice were randomly assigned to experimental groups by drawing lots. The investigator was blinded to group allocation during all experimental procedures and data analysis. Mast cell distribution was analyzed using Toluidine Blue staining to confirm the Wsh functional model. The animals were obtained from the Research Unit for the Production of Laboratory Animals at CINVESTAV-IPN in Mexico City. The mice had unrestricted access to standard rodent food and water *ad libitum*. Animals were cared for and handled according to the International Review Board Regulations and the Mexican Animal Protection Law NOM-062-ZOO-1999. This study was approved by the Bioethics Committee of the School of Medicine, Universidad Autónoma de Nuevo León (approval Number: IN230001).

To develop experimental actinomycetoma, a bacterial cell suspension of *N. brasiliensis* ATCC 700358 was prepared according to the protocol published by Salinas *et al* [2]. Briefly, *N. brasiliensis* bacteria were cultured in brain-heart infusion (BHI) broth (Difco, BD Biosciences) at 37°C for 72 hours until they reached the logarithmic growth phase. The biomass was then collected, washed with sterile saline solution, and homogenized to dissociate the bacterial aggregates. After an additional wash, the residual aggregates were removed via centrifugation at 1,600 rpm for 5 minutes. The supernatant containing a single-cell suspension was collected and adjusted to a concentration of 1× 10^6^ bacteria in 0.05 mL saline solution. Six mice from the *Kit^W-sh/W-sh^* and control groups were infected by subcutaneously inoculating 0.05 mL of the bacterial suspension into the hind footpad. The mice were assessed for clinical signs of inflammation and somatometry until 72 days post-infection (DPI), when the animals were euthanized via ketamine/xylazine (100/10 mg/kg) intraperitoneal overdose. After mycetoma formation, the bacterial load was calculated again by replating the infected tissue in BHI agar and counting the number of colony-forming units per gram of tissue. Our experiments were performed in 2 independent replicates.

### Histopathological Analysis

At 30 and 72 DPI, mice were euthanized to obtain intact mycetoma lesions through incisions at the level of the tibiofemoral joint. The mycetoma was fixed in 10% neutral buffered formalin. The tissue was embedded in paraffin and sliced into 4 µm thick sections prior to histochemistry (Hematoxylin/Eosin, Toluidine Blue-staining). All staining was performed at room temperature. Whole-slide scans were performed using a DP200 slide scanner (Cat. no. 08303916001; Roche Tissue Diagnostics; Roche Diagnostics, Ltd.), followed by morphometric analysis using QuPath v0.5.1 [8]. The identification of *Nocardia* grains in mycetoma is based on their characteristic morphological features. We evaluated 1) the number of bacterial grains/area and 2) the area occupied by the grains in square micrometers.

### Quantification of Anti-*Nocardia* IgG Antibodies by ELISA

To evaluate the presence of anti-*Nocardia* antibodies, we performed a sandwich-type immunosorbent assay using 96-well polystyrene plates. As a capture antigen, we used 0.5 μg per well of the purified 24-kDa protein from *N. brasiliensis*, which was incubated overnight at 4°C. After 3 washes with PBS-Tween 20 (1:1000, pH 7.2), the plate was blocked with 5% skim milk in PBS. The plate was washed again and incubated at 37°C for 1 hour with the mouse serum diluted 1:500. After washing, 200 µL/well of goat anti-mouse immunoglobulin G (IgG) conjugated to peroxidase was added and incubated for 1 hour at 37°C. The chromogen substrate solution (o-phenylenediamine + hydrogen peroxide) was added, and 1N sulfuric acid was used as a stop reagent. The absorbance at 492 nm was analyzed using a semiautomated ELISA plate reader (Stat Fax 3200).

### Flow Cytometry

For cytometric staining, single-cell suspensions were obtained from the footpad lesions via enzymatic digestion. Briefly, the tissue was mechanically and enzymatically digested in DMEM supplemented with 5% FBS (cat. no. F2442; SigmaAldrich) and an enzymatic cocktail containing collagenase (cat. no. C9891; MilliporeSigma), hyaluronidase (cat. no. H3506; MilliporeSigma), DNase (no. 10104159001; Roche Diagnostics, Ltd.), trypsin (cat. no. 15090046; Gibco; Thermo Fisher Scientific, Inc.), and EGTA (cat. no. E3889; MilliporeSigma). The cell suspension was stained with APC-Cy7 mouse anti-CD3 (BD, clone 17A2) and PE mouse anti-Ly6G/6C (BD, clone RB6-8C5) antibodies for 30 minutes at 4°C and protected from light. After staining, 100,000 events were acquired using a Fortessa flow cytometer (BD LSR Fortessa, Hercules). Neutrophils (Ly6G/6C^+^) and lymphocytes (CD3^+^) were counted after discarding doublets and cellular detritus using the following flow strategy: FSC-H vs FSC-A > SSC-A vs FSC-A > Ly6G/6C^+^ vs CD3^+^. Cytometric analyses were performed using FACSDiva software v8.0 for Windows (BD Biosciences).

### Mast Cells Culture

Bone Marrow-derived Mast Cells (BMMCs) were differentiated following the protocol previously described by Campillo-Navarro *et al* [9]. Briefly, bone marrow progenitors were isolated from the femur and tibia of 6-8-week-old female C57BL/6 mice. The cells were cultured in complete RPMI-1640 medium supplemented with 10% FBS, 5 mM β-mercaptoethanol (Life Technologies), 2% antibiotic (Sigma), murine recombinant IL-3 (10 ng/mL), and murine recombinant stem cell factor (10 ng/mL; both factors from Peprotech). To promote selective mast cell growth, non-adherent cells were transferred to fresh medium twice weekly for 6–9 weeks. BMMC purity was confirmed by flow cytometry, with ≥90% of cells showing a double-positive phenotype for CD117 (c-Kit, clone 2B8, BioLegend) and FceRI (clone MAR-1, BioLegend). After staining, cell viability was measured by flow cytometry using a FACSAria Fusion (BD Biosciences). Data were analyzed with FlowJo software version 6.0 (FlowJo, LLC). An unstained control was included for autofluorescence compensation.

### Degranulation Assay

BMMCs (2 × 10^5^ cells in 0.25 mL of HEPES-Tyrode buffer [HBT; 130 mM NaCl, 5.5 mM glucose, 2.7 mM KCl, 1.0 mM CaCl_2_·2H_2_O, 0.1% (w/v) bovine serum albumin (BSA), 12 mM HEPES, 0.45 mM NaH_2_PO_4_·1H_2_O, pH 7.2]) were allocated into the following experimental groups: unstimulated (negative control), stimulated with 10 µg/mL compound 48/80 (positive control), or stimulated with *N. brasiliensis* at multiplicities of infection (MOI) of 0.5, 1, or 3. Cells were incubated for 90 minutes at 37°C. Following stimulation, the supernatants were collected by centrifugation. The cell pellet was subsequently lysed with 200 µL of 0.2% Triton X-100 in HBT. To quantify β-hexosaminidase activity, both supernatants (released enzyme) and cell lysates (retained enzyme) were incubated with 1 mM 4-methylumbelliferyl N-acetyl-β-D-glucosaminide (Sigma-Aldrich) in 200 mM sodium citrate buffer (pH 4.5) for 2 hours at 37°C. The enzymatic reaction was terminated by adding 100 µL of 200 mM Tris base (pH 10.7). Fluorescence was measured using a SpectraMax M with excitation at 356 nm and emission at 450 nm. The percentage of β-hexosaminidase release was calculated as follows: % Release = [Supernatan / (Supernatan + (Cellpellet)] × 100.

### Quantification of Inflammatory Cytokines by ELISA

Inflammatory cytokines were analyzed in the culture supernatants 24 hours after *N. brasiliensis* infection. For the assay, we used 2.5 × 10^5^ BMMCs cultured in RPMI 1640 medium with *N. brasiliensis* at MOI of 0.5, 1.0, and 3.0. The cytokines IL-6, TNF-α, and IL-1β were assayed using a commercial ELISA kit following the manufacturer’s instructions (BioLegend). Absorbance was measured using a Multiskan FC 357 Microplate Photometer (Thermo Fisher Scientific) and SkanIt v6.1.1.7 software.

### Statistical Analysis

After assessing normality using the Shapiro-Wilk test, comparisons between the 2 groups were analyzed using the unpaired t-test. Comparisons between multiple groups were analyzed using the Kruskal-Wallis test or Analysis of Variance, followed by Dunn’s post hoc tests or Tukey’s tests, for non-parametric and parametric data respectively. Statistical significance was set with a *P*-value < 0.05.

## RESULTS

### Mast cells are Essential for the Immunopathological Containment of *N. brasiliensis* in Experimental Actinomycetoma

**Figure 1.**
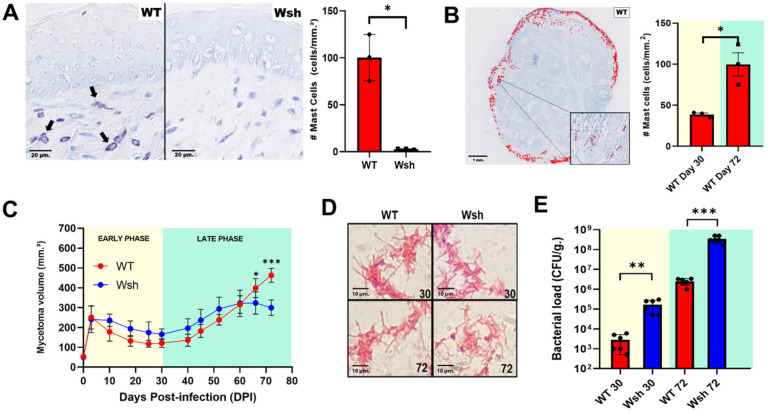
**Mast cells sustain the inflammatory control of *Nocardia brasiliensis* infection in mice with experimental actinomycetoma.** (A) Wsh mice (right panel) had fewer mast cells than WT control mice (left panel). Arrows indicate the presence of Toluidine Blue-positive mast cells. (B) Mast cells localize to the dermis in wild-type (WT) mice with experimental actinomycetoma (inset shows red-circled Toluidine Blue-positive cells). (C) Wsh mice showed decreased mycetoma volume over 72 days of *N. brasiliensis* infection. (D) Kinyoun stain confirms the etiologic agent *N. brasiliensis* at the same days post-infection (DPI) in both actinomycetoma models. (E) Mycetoma lesions in Wsh mice had increased bacterial loads during early and late infection compared to control littermates. **Data are presented as mean ± standard error of the mean. For longitudinal data (C), statistical analysis was performed using 2-way repeated-measures ANOVA with a mixed-effects model (REML) followed by Šídák’s post hoc test. For single time-point comparisons (A, B, E), an unpaired 2-tailed Student’s t-test was used. *P < 0.05, ** *P* < 0.01, * *P* < 0.001; ns, not significant. n = 3-6 mice per group. Each experiment was performed with at least 2 independent biological replicates. CFU = colony-forming units.

Initially, we evaluated the role of mast cells in the clinical evolution of experimental actinomycetoma in mice, a role that has not been previously characterized. To this end, we replicated the experimental model of actinomycetoma based on subcutaneous inoculation of bacteria in mast cell-deficient C57BL/6 *Kit^W-sh/W-sh^* (Wsh) mice and WT C57BL/6 littermates. *Kit^W-sh/W-sh^* mice had a decreased number of tissue mast cells, as observed by Toluidine blue staining (Figure 1A). In control mice, mast cells localize to the dermis and subcutaneous tissue, surrounding the granulomatous core of lesions (Figure 1B). The clinical progression of the experimental mycetoma did not show differences in early infection until 68 DPI, where *Kit^W-sh/W-sh^* mice presented smaller lesions, indirectly suggesting decreased tissue inflammation (Figure 1C). Viable *N. brasiliensis* was successfully isolated and cultured from actinomycetoma lesions in both mice models. Bacterial phenotype was confirmed by Kinyoun staining, which demonstrated the maintenance of viable bacilli through both the early and late stages of infection (Figure 1D). Complementary to size, the lesions showed increased bacterial burden in *Kit^W-sh/W-sh^* mice throughout the late phase of infection compared with control mice (Figure 1E). Collectively, our results suggest that mast cells play an important role in controlling *N. brasiliensis* infection, not during acute infection but in the late, chronic stage of mycetoma formation.

### The Grains Inside Actinomycetoma Had Structural Differences in *Kit^W-sh/W-sh^* Mice Compared to Control Mice

**Figure 2.**
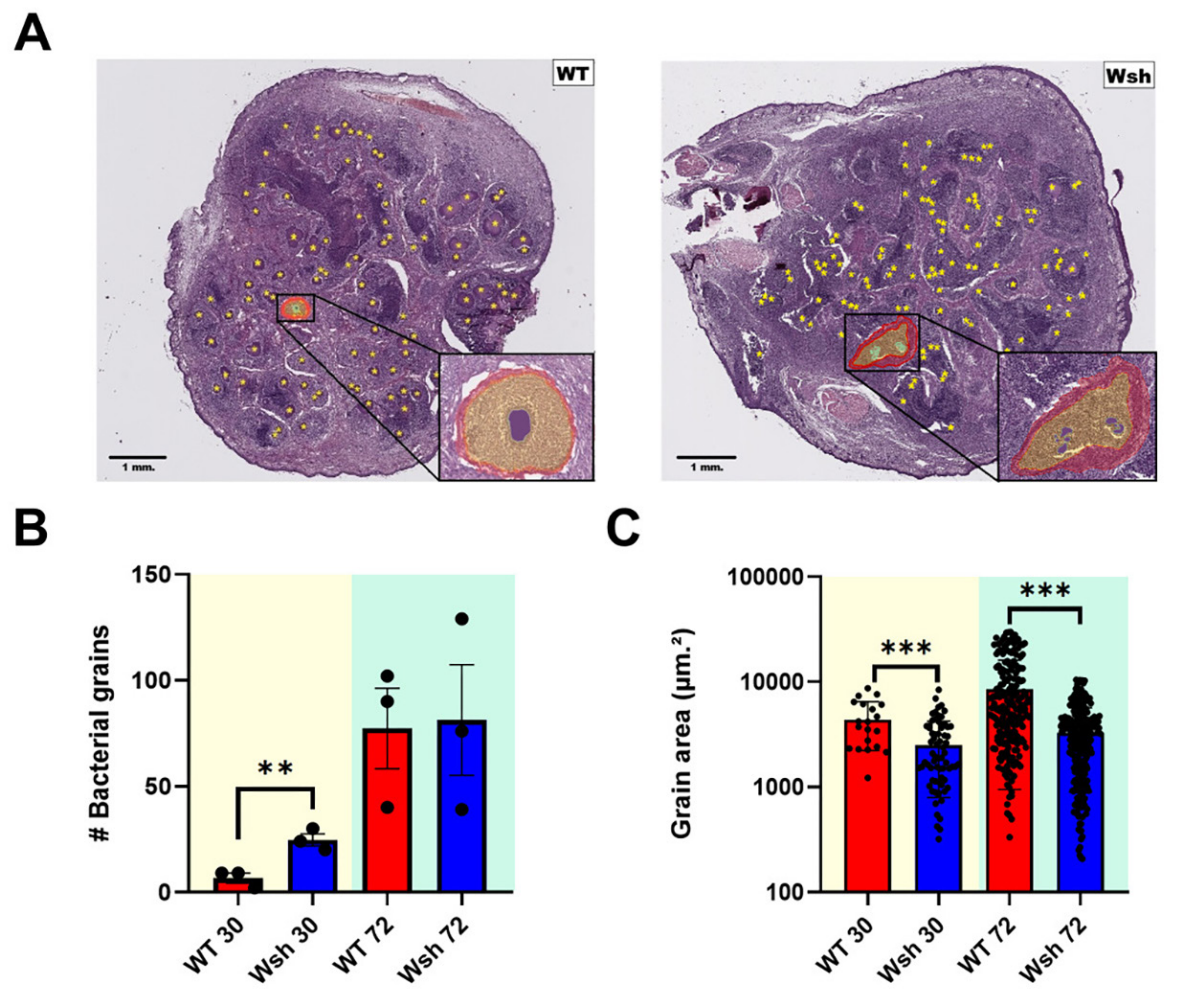
**Mycetoma in Wsh mice showed morphological changes in the bacterial grain structure compared to wild-type (WT) mice**. (A) Whole mycetoma lesions showing the distribution of single grains (yellow dots) in WT (left panel) and Wsh (right panel) mice at 72 days post infection (DPI). The inserts show the typical granuloma structure: a central grain surrounded by polymorphonuclear cell infiltrates (yellow mask) and a fibrotic ring (red-colored tissue). Mycetoma at 72 DPI. Hematoxylin and eosin staining. (B) In Wsh mice, mycetoma lesions exhibited an increase in bacterial grains at 30 DPI, which then stabilized or reverted by 72 DPI. (C) Wsh mice exhibited smaller bacterial grains compared to those in WT mice. n = 3 mice. Total bacterial grains in each mycetoma lesion were counted, and the area of each individual bacterial grain was measured. Data are presented as mean ± standard error of the mean. Comparisons between WT and Wsh mice were performed separately for each time point using unpaired ttest. No comparisons were made between time points. **P < 0.01, *** *P* < 0.001.

In addition to the changes in volume and bacterial burden in mycetoma, we examined the distribution and morphological characteristics of grains in both *Kit^W-sh/W-sh^* and control mice (Figure 2A). The number of bacterial grains increased in *Kit^W-sh/W-sh^* mice at 30 DPI, leveling off thereafter (Figure 2B). In contrast, the average grain area was smaller in *Kit^W-sh/W-sh^* mice than in control littermates (Figure 2C). Overall, the trend toward an increased number of smaller bacterial grains (decreased area) suggests abnormal maturation of the bacterial microcolonies and subtle difficulties in establishing a robust infection.

### The Experimental Mycetoma in *Kit^W-sh/W-sh^* Mice Showed an Initial Numerical Advantage in Myeloid Cell Infiltration Accompanied by Transient Splenic Enlargement, in Comparison to Control Mice

**Figure 3.**
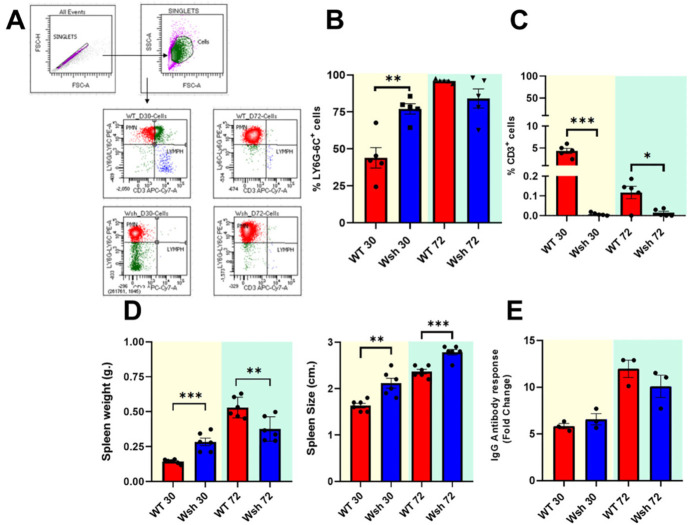
**Wsh mice showed decreased leukocyte infiltration and increased splenic size after experimental mycetoma with *Nocardia brasiliensis.*** (A) Representative flow plots for leukocyte infiltration in mycetoma show a predominance of Ly6G/6C+ polymorphonuclear cells. (B) Mycetoma in Wsh mice showed an initial numerical advantage in polymorphonuclear cells at 30 days post infection (DPI), followed by a failure to sustain this response at 72 DPI, whereas control mice exhibited sustained infiltration over time. (C) Mycetoma in Wsh mice showed fewer lymphocytes compared to that in control mice on the same days post-infection. n = 5 mice per group. (D) Wsh mice showed increased splenic weight (left panel) and size (right panel), n = 6 mice per group; each experiment was performed with at least 2 independent biological replicates. (E) Wsh mice showed similar titers in antibody response to *N. brasiliensis* relative to wild-type (WT) mice, n = 3 mice per group. Data are presented as mean ± standard error of the mean. Comparisons between WT and Wsh mice were performed separately for each time point using unpaired ttest. No comparisons were made between time points. *P < 0.05, **P < 0.01, ***P < 0.001. Fold change relative to non-infected littermates.

We quantified the leukocyte cellularity within the active lesion and other parameters to identify systemic changes associated with clinical mycetoma in the absence of mast cells. Infiltrating leukocytes were evaluated using flow cytometry after enzymatic digestion of active lesions (Figure 3A). Mycetoma in *Kit^W-sh/W-sh^* mice showed an initial numerical advantage in Ly6G/6C^+^ neutrophils at 30 DPI, a finding consistent with the baseline extramedullary myelopoiesis characteristic of this strain, but this advantage was not sustained, as control mice surpassed *Kit^W-sh/W-sh^* mice by 72 DPI (Figure 3B). Likewise, mycetoma in *Kit^W-sh/W-sh^* mice showed a lower percentage of CD3^+^ infiltrating lymphocytes than that in control mice (Figure 3C). Among systemic changes, the spleen weight and size were increased in *Kit^W-sh/W-sh^* mice relative to control mice at 30 DPI, but this difference was no longer evident at 72 DPI, mirroring the pattern observed in the myeloid infiltrate (Figure 3D). We then examined the adaptive humoral response by assessing the levels of circulating anti-*Nocardia* IgG. The results indicated similar levels of circulating antibodies in *Kit^W-sh/W-sh^* and control mice (Figure 3E). Collectively, the decreased number of infiltrating lymphocytes, in addition to the associated splenomegaly, may suggest a dissociated inflammatory state between the local lesion and systemic inflammatory signs.

### Inflammatory Activation of BMMCs After *In Vitro* Infection With *N. brasiliensis*

**Figure 4.**
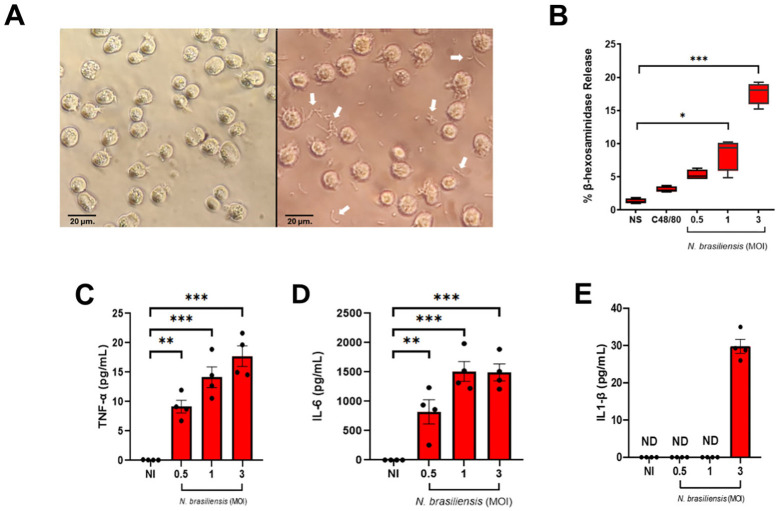
***In vitro* infection with *Nocardia brasiliensis* induces the synthesis of inflammatory mediators in bone marrow-derived mast cells (BMMC)**. (A) Representative micrographs (x100) of infected (right panel) and control (left panel) mast cell cultures. Arrows indicate extracellular bacteria. (B) *N. brasiliensis* induced degranulation in cultured BMMCs. Degranulation was determined through beta-hexosaminidase release at the MOI indicated. *N. brasiliensis* increased the synthesis of TNF-α (C), IL-6 (D), and IL-1β (E) in cultured BMMCs. Data are presented as median ± interquartile range (B) and mean ± standard error of the mean (C-E). Kruskal-Wallis (B) and ANOVA (C-E) tests were used. *P < 0.05, ** *P* < 0.01, *** *P* < 0.001. n=4 mice, NS= Non-Stimulated, NI = Non-Infected.

After observing that mast cells play a relevant role in the experimental model of actinomycetoma, we decided to evaluate whether mast cells alone are capable of recognizing and becoming activated in response to *N. brasiliensis*. For this purpose, we infected BMMCs using an *in vitro* approach with a *N. brasiliensis*. As depicted in (Figure 4A), bacterial cells had tight contact with mast cells through their plasma membrane, leading to bacterial aggregation. Based on previous work, we used a multiplicity of infection (MOI) of 3 [10]. We next sought to determine whether the observed direct contact between mast cells and *N. brasiliensis* led to effector activation. Therefore, we assessed the degranulation level of BMMCs following infection. The release of β-hexosaminidase, a classic marker of mast cell granule exocytosis, was quantified in the culture supernatants. As shown in Figure 4B, infection with *N. brasiliensis* at an MOI of 3 induced significant degranulation in BMMCs compared to unstimulated cells (basal control). This increase in β-hexosaminidase release was dose-dependent, with a stronger response observed at MOI 3 compared to MOI 1.

These results demonstrate that *N. brasiliensis* not only establishes physical contact with mast cells but is also a sufficient stimulus to trigger their secretory response. Next, we analyzed the effect of *in vitro N. brasiliensis* infection on the synthesis of inflammatory cytokines by cultured BMMCs. We found increased levels of the cytokines tumor necrosis factor alpha (TNF-α) and interleukin-6 (IL-6) for MOIs from 0.5 to 3, but in contrast, IL-1β required an MOI of 3 to be detectable (Figure 4C-E). These results suggest that mast cells can be directly activated by *N. brasiliensi*s, primarily through the detection of bacterial-associated molecular patterns, thereby inducing the release of various inflammatory mediators.

## DISCUSSION

The role of mast cells as sentinel cells of the innate immune system is well established, owing to their strategic anatomical localization at host–environment interfaces. Given that *N. brasiliensis* infection breaches physical barriers such as skin, defining the specific role of mast cells in this context is of considerable interest. To investigate this, we employed a mast celldeficient murine model (*Kit^W-sh/W-sh^*). Unlike other classical *Kit* mutants, *Kit^W-sh/W-sh^* mice are neither anemic nor sterile and maintain largely normal hematopoiesis [11], though nonmast cell abnormalities have been described in this strain and are considered below. In WT mice, mast cells localized to the periphery of granulomas (Figure 1B), similar to *Mycobacterium tuberculosis* granulomas [12, 13]; this mast cell topographical distribution suggests a host immune response to contain the bacterial spread. In *Kit^W-sh/W-sh^* mice infected with *N. brasiliensis*, we observed reduced inflammation and increased bacterial burden, thus suggesting a relation between inflammation and bacterial load. The absence of mast cells is directly associated with an increase in colony-forming units, similar to tuberculosis [7] and *Borrelia burgdorferi* [14], and contrasts with the dispensable role of mast cells in early wound healing [15]. This highlights their specific involvement in persistent disease [12, 16]. Notably, *Kit^W-sh/W-sh^* mice retained some features of granuloma, including myeloid infiltration and a peripheral fibrotic ring. However, unlike the wellorganized, ovoid granulomas observed in WT mice, lesions in mast celldeficient animals exhibited distinct features that led us to classify them as disorganized. Specifically, these lesions were structurally irregular (nonovoid), lacked a compact architecture, and contained multiple bacterial grains (>2 per lesion) within a single infectious focus, in contrast to the single or few wellformed grains typical of WT granulomas (Figure 2A). Based on these morphological criteria—irregular shape, loss of compactness, and multifocal bacterial distribution—we consider these lesions disorganized granulomas. This suggests that mast cells are not absolutely required for the recruitment of inflammatory cells or the deposition of collagen, but they could contribute to the spatial organization of the granuloma and to containing bacterial proliferation into a single, welldefined focus. While these data suggest a contribution from mast cells in granuloma organization and bacterial containment, they do not definitively prove mast cell-specific causality, as the *Kit^W-sh/W-sh^* strain has been reported to harbor additional abnormalities that could contribute to the observed phenotype.

Analysis of myeloid cell recruitment revealed that *Kit^W-sh/W-sh^* mice exhibited an initial numerical advantage at 30 DPI, likely due to baseline neutrophilia and aberrant extramedullary myelopoiesis [17]. Although Ly6G/6C does not definitively distinguish neutrophils from monocytes, flow cytometry characterization in this murine model confirmed that >90% of Ly6G/6C^+^ cells in infected tissue are neutrophils [3]. Despite this advantage, proper granuloma organization was not achieved, and by 72 DPI, WT mice surpassed *Kit^W-sh/W-sh^* mice in myeloid cell numbers, indicating a failure to sustain the myeloid response in the absence of mast cells. This pattern was mirrored in splenic cellularity [18], reinforcing the existence of a hypothetical “critical period” early in infection during which the failure to sustain myeloid recruitment in Wsh mice may impair subsequent granuloma maturation [19, 20]. However, while our data are consistent with a role for mast cells in sustaining myeloid recruitment, they do not directly demonstrate that mast cellderived mediators are responsible for these findings. We cannot exclude that other *Kit^W-sh/W-sh^*-associated abnormalities contribute to this phenotype. Additional experiments are needed to understand *in vivo* levels of mast cellassociated cytokines (TNFα, IL6, IL1β) directly in infected tissue, as well as the neutrophil chemoattractants CXCL1 and CXCL2. We also observed a significant deficit in CD3^+^ T lymphocyte recruitment (<5% of lesional infiltrate), consistent with previous reports from our group demonstrating Th1 and Th17 cells in the actinomycetoma microenvironment [4, 21]. However, in Wsh mice, apparently in the absence of mast cells, both the inflammatory phase (Ly6G/6C^+^ neutrophils) and the subsequent regulatory and adaptive cellular response (T cells) are compromised, leading to dysregulation of the typical immune response in this model [22]. Our results showing a decreased T cell percentage in infected tissue may reflect a direct relation of lack of mast cell number in Wsh mice; however, nonmast cell abnormalities that may affect T cell function independently of mast cell deficiency deserve further investigation. Systemic observations, including splenic dysregulation alongside an intact antiNocardia IgG response, indicate that the humoral adaptive response remains functional, whereas the impact of mast cell deficiency appears confined to a localized defect in tissueorganizing cellular immunity.

To directly test mast cell activation by the pathogen, we employed an *in vitro* system using BM-MCs. Coculture assays demonstrated direct interaction and robust activation (Figure. 4C), with dosedependent βhexosaminidase release and production of TNFα, IL6, and IL1β. The finding that *N. brasiliensis* induces both degranulation and cytokine production—whereas some pathogens trigger only one pathway—suggests that *N. brasiliensis* may engage multiple receptors on mast cells [22]. The concomitant induction of early cytokines (IL-6, TNF-α) and late, highburdendependent release of IL-1β indicates a biphasic response. These *in vitro* data provide a plausible mechanistic basis for the *in vivo* observations. Importantly, the cytokine profile we observed is consistent with previous reports of TNF-α, IL-1β, and IL-6 in *N. brasiliensis* infected dermis [23]; our data now identify mast cells as one likely contributor to this early cytokine milieu.

The triad of cytokines released by activated mast cells aligns with established mechanisms of neutrophil recruitment. IL-1R signaling is essential for neutrophil recruitment in cutaneous *Staphylococcus aureus* infection [24]. TNF-α promotes endothelial adhesion and enhances neutrophil effector functions [25, 26]. IL-6 regulates neutrophil trafficking via CXCL1/KC through STAT3 [27]. Thus, mast cell-derived cytokines may contribute to neutrophil recruitment and activation, with neutrophils serving as a structural and immunological cornerstone within the *N. brasiliensis* granuloma. Previous work from our group has demonstrated sustained elevation of systemic IL-6 during chronic actinomycetoma [3], reinforcing the concept that mast cell-derived signals may contribute to an immune equilibrium favoring bacterial persistence.

Several limitations warrant consideration. First, while the *Kit^W-sh/W-sh^* mice are widely used for mast cell functional analysis, they harbor a mutation in the *cKit* regulatory element that may affect other cell types, including melanocytes, interstitial cells of Cajal, and potentially T-cell populations. These non-mast-cell abnormalities could account for some of the observed outcomes, particularly the deficit in T cell recruitment. Thus, our findings support but do not definitively prove mast-cell-specific causality. Second, our *in vitro* BMMC system lacks the complex tissue microenvironment that modulates mast cell behavior *in vivo*. Third, the specific molecular receptors (TLRs, C-type lectins, or others) involved in *N. brasiliensis* recognition by mast cells remain to be identified. Including a heat-killed bacteria control in future studies would help determine whether activation is driven by specific PAMPs—thereby informing which receptors may be involved—or by nonspecific phagocytosis of live bacteria.

## CONCLUSION

In conclusion, our data indicate that mast cells are activated by *N. brasiliensis* and contribute to granuloma organization and bacterial containment in experimental actinomycetoma, though definitive causality requires confirmation with mast cellspecific reconstitution or selective depletion models. We propose that mast cells serve as contributors of the granulomatous response, with a participatory role in sustaining myeloid recruitment during a hypothetical “critical period” that defines the architecture of the infectious niche and the host’s ability to contain chronic infection.

## LIMITATIONS:

This study utilized the *Kit^W-sh/W-sh^* mouse model to establish a foundational link between mast cell deficiency and the progression of experimental *N. brasiliensis* mycetoma. A more detailed mechanistic dissection of how mast cells precisely regulate late-stage inflammation, neutrophil recruitment, and bacterial grain formation was beyond our present scope. Future work is required to determine whether specific mast cell-derived mediators—such as proteases, TNF-α, or specific cytokines—directly orchestrate these processes. The experimental model established here provides a controlled system for testing targeted interventions, including mast cell stabilizers, mediator-specific antagonists, and selective anti-inflammatory therapies. These studies may yield critical insights into the immunopathology of mycetoma and could inform future strategies for modulating the chronic inflammation characteristic of this disease.
